# Snake Venom PLA_2_ as Anticoagulant Agents: Role of Crotoxin, from *Crotalus durissus* Rattlesnake, in Hemostasis

**DOI:** 10.3390/toxins17120583

**Published:** 2025-12-05

**Authors:** Lisele Maria Brasileiro-Martins, Greene Dias Marques, Jéssica Burlamaque Maciel, Márcia Neiva, Thaís Pinto Nascimento, David Jose Estrada Reyes, Alessandro Júnio Campelo Feitosa, Sofia Angiole-Cavalcante, Priscila Ferreira de Aquino, Jacqueline de Almeida Gonçalves Sachett, Wuelton Marcelo Monteiro, Marco Aurélio Sartim

**Affiliations:** 1 Postgraduate Program Biodiversity and Biotechnology Network, Amazonas State University, Manaus 69065-001, Brazil; liselebrasileiro@hotmail.com (L.M.B.-M.); neivamarcia2017@gmail.com (M.N.); 2 Department of Teaching and Research, Fundação de Medicina Tropical Dr. Heitor Vieira Dourado (FMT-HVD), Manaus 69040-000, Brazil; jessicabmaciel@hotmail.com (J.B.M.); thaispinto.am@gmail.com (T.P.N.); davidj.estradar@gmail.com (D.J.E.R.); jac.sachett@gmail.com (J.d.A.G.S.); wueltonmm@gmail.com (W.M.M.); 3 Vice-Rectorate for Research and Graduate Studies, Nilton Lins University, Manaus 69058-030, Brazil; 4 Normal Superior School, Amazonas State University, Manaus 69050-010, Brazil; greenedias@gmail.com; 5 Postgraduate Program in Tropical Medicine, Amazonas State University (UEA), Manaus 69050-001, Brazil; 6 Postgraduate Program in Pharmaceutical Sciences, Federal University of Amazonas (UFAM), Manaus 69080-900, Brazil; sandrojunio19@gmail.com; 7 Leonidas and Maria Deane Institute, Oswaldo Cruz Foundation, Manaus 69057-070, Brazil; s.angiole.c@gmail.com (S.A.-C.); priscila.aquino@fiocruz.br (P.F.d.A.); 8 Duke Global Health Institute, Duke University, Durham, NC 27710, USA

**Keywords:** *Crotalus durissus*, crotoxin, anticoagulant, hemostasis, phospholipases A_2_, coagulation cascade

## Abstract

Snake venoms are rich sources of bioactive molecules that modulate hemostasis and, among these, anticoagulant snake venom phospholipases A_2_ (sPLA_2_) are found in a range of snake venoms. Crotoxin (CTX), from the *Crotalus durissus* rattlesnake, is a heterodimeric PLA_2_ complex, and literature has reported its mechanisms in anticoagulant activity. The present review revisits the biological roles of anticoagulant sPLA_2_ and critically examines evidence on CTX in hemostatic regulation, aiming to clarify its mechanisms and therapeutic promise. CTX exerts anticoagulant activity via enzymatic hydrolysis of procoagulant phospholipids and direct interaction with coagulation factors, disrupting key complex assembly. It also counteracts inflammation-induced coagulation by modulating leukocyte- and endothelial-derived mediators, restoring balance among anticoagulant, procoagulant, and fibrinolytic pathways. Effects on platelet function appear comparatively modest, ranging from less potent pro-aggregatory activity to negligible aggregation. The dual anticoagulant and anti-inflammatory properties of CTX highlight its potential as a model for novel antithrombotic agents in hypercoagulable and inflammation-driven disorders, despite toxicological concerns that necessitate cautious pharmacological exploration.

## 1. Introduction

The hemostatic alterations caused by snake venoms result from biologically active toxins that can trigger or suppress blood coagulation, platelet function, and fibrinolysis. Although these effects are contrasting, they ultimately lead to the same clinical outcome—bleeding [1]. Anticoagulant phospholipases A_2_ from snake venoms (sPLA_2_) represent a group of toxins that impair the blood coagulation process. These enzymes are classified according to molecular and functional share and are highly prevalent in the venoms of snakes from the Viperidae family. Research has focused on their roles during envenomation and on their potential as sources of novel anticoagulant drugs [2].

**Figure 1:**
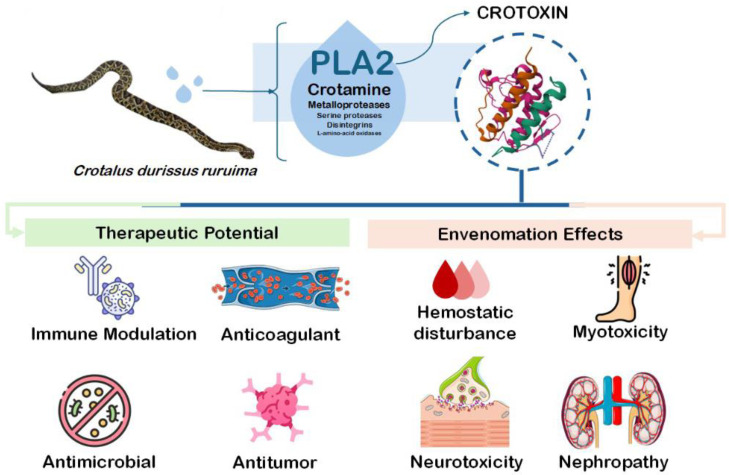
Toxicological and pharmacological properties of Crotoxin.

The rattlesnake *Crotalus durissus* is an important species in South America and represents one of the major groups of medically significant snakes in the region [3]. Among the various components of its venom, crotoxin (CTX) consists of a phospholipase A_2_ enzymatic complex that induces marked systemic myotoxicity, neuromuscular blockade, and nephropathy [4,5]. In addition to these effects, studies have shown that crotoxin also modulates hemostasis by inhibiting coagulation activation, altering platelet function, and disrupting inflammation-mediated thrombo-coagulation [6,7,8]. Aside from hemostasis, CTX have been demonstrated to present different pharmacological relevance as antitumoral, antimicrobial, and immunomodulatory behavior [9,10,11] (Figure 1). As the principal component of *C. durissus* venom, CTX’s role in hemostasis highlights not only its contribution to bleeding disorders during envenomation but also its potential value for bioprospecting novel molecules.

The present review aims to provide a comprehensive overview of anticoagulant sPLA_2_ mechanism on hemostasis and uncover the effects and mechanisms of CTX on hemostasis, highlighting its potential for therapeutic applications or as a molecular tool for laboratory testing within hemostasis diseases.

## 2. Role of Snake Venom Phospholipases A_2_ in Hemostasis: The Anticoagulant SVPLA_2s_

Snake venom phospholipases A_2_ (sPLA_2_) are among the most widely distributed and functionally diverse components of snake venoms, occurring across different snake families. These enzymes belong to Group I and II secreted phospholipases, with the capacity to hydrolyze the sn-2 ester bond of glycerophospholipids present in biological fluids or cell membranes, thereby releasing free fatty acids and lysophospholipids [12]. These enzymes can be categorized into two groups, regarding enzymatic activity: (I) Asp49 sPLA_2_s are enzymes that contain a conserved aspartic acid (Asp) residue at position 49 in the catalytic site, which confers greater structural stability to the calcium-binding region and is consequently essential for catalytic activity; and (II) the non-Asp49 SVPLA_2_s, also known as ‘sPLA_2_-like’ proteins or ‘Lys49 sPLA_2_ homologues,’ are enzymes that share high sequence and structural similarity with Asp49 sPLA_2_s but contain a lysine or other residue in place of the conserved Asp49, a substitution that results in minimal or no catalytic activity [13]. The sPLA_2_s perform a range of biological functions and exert various pharmacological effects, including neurotoxicity, myotoxicity, hemolysis, disrupting hemostasis events, and modulating inflammatory responses [14,15,16,17].

Anticoagulant sPLA_2_ enzymes are generally classified as either weak or strong anticoagulants, and their basic character often exhibits stronger anticoagulant activity, although this is not universally true [18]. Along with coagulation, sPLA_2_s also interfere with targets associated with platelet function. The anticoagulant properties of sPLA_2_s was first reported in a 1972 study by Boffa and colleagues (1972) [19]. In this publication, the authors describe the isolation of a sPLA_2_ from the venom of European *Vipera berus*, presenting anticoagulant activities [19]. Since then, numerous anticoagulant sPLA_2_s have been identified and isolated from the Viperidae, Crotalidae, Elapidae, and Hydrophiidae families, presenting diverse structural and functional characteristics [20]. Studies have demonstrated that two main mechanisms were involved in the anticoagulant activity, consisting of enzymatic hydrolysis of procoagulant phospholipids and/or a direct protein–protein interaction of the toxin with blood coagulation factors (independently of catalytic function).

Regarding platelets, the earliest studies in this area focused primarily on evaluating platelet lysis and surface phospholipid cleavage induced by sPLA_2_ [21]. However, the first evidence on the interference with platelet functionality was reported by Chap and colleagues (1977) [22], showing that a sPLA_2_ isolated from *Naja naja* venom could induce platelet aggregation [22]. To date, various other SVPLA_2_s have been shown to induce or inhibit platelet aggregation, with mechanisms involving formation of arachidonic acid-derived products from sPLA_2_ phospholipid catalysis [23]. Regarding anticoagulant sPLA_2_s, not all can modulate platelet function; however, they share the same mechanisms mentioned.

**Figure 2:**
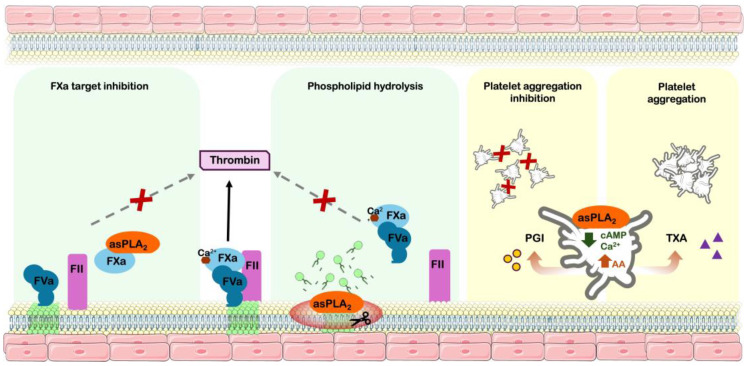
Hemostatic modulation mechanisms of snake venom phospholipases A_2_. Pathophysiology pathways of snake venom phospholipases A_2_; inducing anticoagulant effect: coagulation factor Xa inhibition and procoagulant phospholipids hydrolysis. Modulation of platelet function: inhibitory effect by arachidonic acid derived prostaglandin I2 production and decreasing intracellular cAMP/calcium and platelet aggregation effect by production of arachidonic acid-derived thromboxane A_2_. asPLA_2_—anticoagulant sPLA_2_; FXa—activated coagulation factor X; FVa—activated coagulation factor V; FII—Coagulation factor II (prothrombin); PGI—prostaglandin I2; AA—arachidonic acid; TXA—Thromboxane A_2_; cAMP—Cyclic AMP; green phospholipids—procoagulant phospholipids.

Over the years, various studies have described multiple mechanisms by which anticoagulant sPLA_2_s interfere with hemostasis, including the cleavage of pro-coagulant phospholipids, direct binding to coagulation factors or complexes, and modulation of platelet function (Figure 2).

### 2.1. The Phospholipid Hydrolysis Mechanism

Phospholipids (PL) are essential for the proper functioning of the coagulation cascade. Upon platelet activation or cell damage, PLs are responsible for creating a negatively charged environment outer membrane surface, crucial for the binding and activation of various coagulation factors. This surface facilitates the assembly of both intrinsic (factors VIIIa/IXa) and extrinsic tenase (factors III/VIIa) and prothrombinase (factors Va/Xa) complexes, which are essential for the generation of thrombin and subsequent fibrin clot formation [24].

The anticoagulant sPLA_2_s are classified into strong, moderate, and weak anticoagulants, depending on the dose needed to induce plasma coagulation (a lesser amount equals increased potency). The hydrolytic activity of anticoagulant sPLA_2_s on phospholipids (PLs) has been associated with one of its anticoagulant mechanisms, as evidenced by various isoforms isolated from a broad range of snake species globally [20]. Their affinity for procoagulant phospholipids (such as phosphatidylserine), rather than other PLs, and enzymatic efficiency underlie their anticoagulant potency. While strong anticoagulant sPLA_2_ are capable of preventing plasma coagulation with only residual hydrolysis of specific plasma phospholipids, weak aSVPLA_2_s need much more hydrolysis in order to show delayed clotting [25].

Beyond phospholipid specificity, another critical mechanistic determinant of anticoagulant sPLA_2_ anticoagulant potency is their “penetration ability”—the capacity of the enzyme to insert into and hydrolyze membrane phospholipids, especially those expressed on the surface of activated platelets or derived micro-vesicles. The ability of sPLA_2_ to penetrate membranes has been identified as a key determinant of anticoagulant efficacy, as this property markedly enhances phospholipid hydrolysis [12].

### 2.2. The Coagulation Factor Complex Inhibition

Although the ability of anticoagulant sPLA_2_s to mediate disruption/degradation of phospholipid surfaces, and to interfere with the assembly of various coagulation complexes, the hydrolysis mechanism itself is only responsible for a mild/moderate anticoagulant potency, independently of strong or weak anticoagulant isoforms. Based on initial controversial results showing that, for some anticoagulant sPLA_2_s there was no correlation between catalytic activity and anticoagulant effects [26], a different mechanism has been determined based on inhibition of coagulation complexes by a direct protein-binding to coagulation factors [27].

During the coagulation cascade, thrombin generation—and subsequent fibrin clot formation—is driven by the activation of prothrombin via the prothrombinase complex, composed of coagulation factors Xa and Va assembled on a phospholipid surface in the presence of calcium. The generation of Factor Xa is significantly initiated through the extrinsic tenase complex, formed by tissue factor (Factor III) and activated Factor VII (VIIa), which operates independently of phospholipids at the onset [28].

Studies using different approaches to inhibit sPLA_2_ catalytic activity (such as chemical alteration of active site, removal of Ca^2+^, or use of modified phospholipids) have demonstrated that anticoagulant sPLA_2_ are still capable of inducing anticoagulant effects. The literature has demonstrated that the noncatalytic mechanism involves the direct binding to blood coagulation factors, impairing the extrinsic tenase complex and/or prothrombinase complex assemblance, binding directly to FXa (but not to Va or IIa) to prevent its association with FVa and inhibit blood clotting [12]. The proposed “target model” demonstrates that specific target sites at coagulation factors, such as FXa, can bind to “pharmacological sites” on anticoagulant sPLA_2_s, enabling complex assemblance with other coagulation factors or PLs [27]. The segment of anticoagulant sPLA_2_ associated with their anticoagulant function lies between residues 54 and 77. This region carries a positive charge in enzymes with strong anticoagulant activity whereas, in weak or no anticoagulant effects, it is either neutral or negatively charged. Notably, these latter forms also lack binding affinity for factor Xa (FXa) [2].

### 2.3. sPLA2 and Platelets

Platelets play a vital role in hemostasis by forming a temporary plug through aggregation and serving as a stationary surface for the assembly of the coagulation cascade, facilitating clot formation and preventing excessive blood loss [29]. Snakebites, especially from the Viperidae family, are well known for their venom-induced coagulopathy, and platelet disturbances are considered an important pathophysiological event associated with severe repercussions [30].

Among hemostatically active toxins, several snake venom PLA_2_s have been reported to significantly modulate platelet functions. Based on their aggregation role, these toxins are capable of inducing, inhibiting, or even promoting (biphasic effect) platelet aggregation [2]. The pro-aggregating mechanisms involve the production of arachidonic acid from membrane PL hydrolysis and, as a consequence, the production of agonist metabolites, such as thromboxane A_2_. The inhibition of platelet aggregation involves catalytic and noncatalytic mechanisms, associated with cleavage of products from arachidonic acid metabolism, or interference with cAMP and Ca^2+^ levels, involving a change in platelet morphology. The biphasic effect consists of the capacity of some sPLA_2_ to present both agonistic and antagonistic behavior, depending on the conditions: in low concentration or after a short incubation time with platelets, a pro-aggregation activity is observed, but an inhibitory effect at a high concentration or prolonged incubation time [31].

Figure 2 summarizes the roles of sPLA_2_ in hemostatic events, reflecting the substantial insights provided by the literature regarding their influence on hemostatic processes. This advances the understanding of their role in snakebite envenomation and supports the bioprospecting of snake venom-derived toxins for medicinal or biotechnological applications. Moreover, the behavior of sPLA_2_ on both coagulation and platelets does not demonstrate a correlation; therefore, the literature describes different isolated anticoagulant sPLA_2_ presenting pro- or anti-platelet aggregation behavior [32].

## 3. Crotoxin, the Major Toxin from South American Rattlesnake *Crotalus durissus* Venom: Role on Hemostatic Events

Snakes of the genus *Crotalus*, known as rattlesnakes, belong to the family Viperidae and comprise approximately thirty species in the American continent. In South America, the species *Crotalus durissus* stands out, encompassing subspecies widely distributed [33,34,35]. In general, venoms from the *Crotalus durissus* subspecies are characterized by abundant PLA_2_s, followed by serine proteases, C-type lectin-like proteins, L-amino acid oxidases, metalloproteases, disintegrins, and others [35].

Crotoxin (CTX) is the major component of all subspecies of *C. durissus* venom, ranging from 50 to over 80% of venom toxins [35]. CTX consists of a heterodimeric protein complex, composed of two non-covalently linked subunits: an acidic, non-toxic, and non-enzymatic subunit (CA or crotapotin), and a basic Lys_49_ enzymatic active phospholipase A_2_ (CB) [27,36,37]. Despite its non-enzymatic activity, crotapotin (CA) is essential for the CTX mechanism of action. The CA subunit acts as a chaperone to the CB portion, enhancing the potency of its PLA_2_ activity [31] by avoiding non-specific interactions through blocking the hydrophobic interfacial binding surface of CB [27].

CTX toxicological behavior has been widely investigated, responsible for neuromuscular blockade and myotoxicity. However, other biological activities have brought the toxin into the perspective of pharmacological bioprospection. This includes its anti-inflammatory and immunomodulatory activity, and the literature has shown the toxin’s capacity to modulate the cellular events of both innate and humoral immunity, as well as to produce endogenous pro-resolving mediators [9].

Another promising perspective for CTX relies on hemostasis. *Crotalus durissus* venom is known to induce coagulation disturbances, majorly associated to the presence of serine proteases acting as thrombin-like enzymes inducing clot formation, and C-type lectin-like potent platelet aggregators, leading to a consumption coagulopathy state in patients and envenomation models [3]. However, the capacity of isolated CTX on hemostatic events has been widely described in the literature, showing that the toxin is capable of inhibiting coagulation activation, altering platelet function, and disrupting thrombo-inflammation [6,7,8]. Therefore, here we discuss further CTX effects on hemostasis and its mechanisms.

### 3.1. Anticoagulant Activity

Verheij (1980) [26] was the first to evaluate the activity of isolated CTX from *Crotalus durissus* terrificus on coagulation. In this study, the authors investigated the coagulation behavior of several phospholipases A_2_ from snake venoms—basic, acidic, and neutral—isolated from species of the Viperidae family. The authors used plasma recalcification time assay as the first screening protocol. Crotoxin was evaluated in two formulations: as the whole CTX complex (subunits A + B), as one of the acidic sPLA_2_s (pI = 5.0); and subunit B only, representing a basic and active fraction of the toxin (pI = 9.5). The results showed that, similar to other acidic sPLA_2_s, the CTX complex displayed weak anticoagulant activity. In contrast, the basic fraction exhibited strong anticoagulant activity, also exclusively found strongly in basic sPLA_2_ [26].

Further studies have also demonstrated the ability of CTX (both the complex and its subunits) to modulate plasma coagulation. Sousa and colleagues [38] evaluated the effects of purified toxins from *Crotalus durissus terrificus* on plasma coagulation, including CTX, as the whole complex and its isolated subunits A and B. In the study, the authors performed both the prothrombin time (PT) and the activated partial thromboplastin time (aPTT), the most widely used coagulation assays for assessing alterations in the extrinsic and intrinsic pathways of the coagulation cascade, respectively. All CTX forms were capable of prolonging plasma clotting in both assays, with subunit B being the most pharmacologically potent, followed by the crotoxin complex, and finally subunit A, which exhibited a more limited effect. Interestingly, in terms of effect magnitude, all CTX forms exerted a more pronounced influence on aPTT plasma coagulation, notably by subunit B.

Similar findings were also observed by Gimenez and colleagues [6], and these authors reported that CTX complex was also capable of prolonging both PT and aPTT plasma clotting times, more intensely on aPTT assay. Moreover, the authors have also evaluated the PT and aPTT assay from plasma obtained from whole blood incubated with CTX, which presented identical efficacy compared to the plasma CTX-incubated protocol. This result demonstrated that the toxin retains a marked and specific anticoagulant effect in blood, compared to plasma, even under conditions involving multiple cellular and molecular targets.

Using a different approach, Prezoto and colleagues [39] have also evaluated CTX complex anticoagulant activity using PT and aPTT assay, but using the thrombo-elastometric method. Another difference is that the authors have performed this assay using chicken plasma, known for its factor XII-deficiency. The authors observed that CTX was capable to inhibit aPTT clotting, but not PT, in a dose-dependent manner. The results also showed a higher potency of inhibition using chicken plasma, compared to human, with a significantly increased sensitivity at the nanoscale level [39].

### 3.2. Phospholipid-Dependent Anticoagulant Mechanism

As mentioned, studies have demonstrated that enzymatically active forms of CTX and subunit B are capable of inducing a much stronger anticoagulant behavior, compared to subunit A or chemically modified inactive CTX. Therefore, mechanistical studies have been conducted to evaluate the involvement of CTX-induced impairment of phospholipids role coagulation.

Gimenez and colleagues [6] have conducted a set of experimental protocols in order to evaluate CTX’s capacity to interfere in coagulation complexes (prothrombinase, intrinsic and extrinsic tenase complexes). The authors performed an assay using the Staclot^®^ DRVV kit (Diagnostica Stago), indicated for the confirmation of the presence of lupus anticoagulants in patients’ plasma. The kit is based on FXa activity in plasma, in two different conditions: at low and high levels of PL. CTX was capable of inhibiting PL-FXa activity at a low PL concentration; however, this was less potent compared to plasma clotting activity. However, at a high PL condition, CTX was not capable of impairing PL-FXa activity. It is important to mention that the authors have also demonstrated that CTX showed no effect on the activity of FXa alone. Therefore, results demonstrate that the toxin is responsible for a disruption of PL-mediated FXa activity; however, it is sensitive to higher amounts of PL [6]. The authors have also evaluated the interference of CTX on intrinsic tenase complex activity (FIX, FVIII, Ca^2+^, and PL), demonstrating a dose-dependent inhibition of the complex. Considering that the authors have not investigated this activity in a PL-independent manner, it is difficult to confirm if the compromising complex formation was dependent on PL disruption or a possible direct binding to coagulation factor IX and/or VIII [6].

Considering that the intravascular coagulation process relies on coagulation complexes anchoring on the PL surface to assemble efficiently, in vitro assay protocols using PL monolayer are more reliable in representing the kinetics of the coagulation process. Verheij and colleagues [26] used the monolayer protocol in order to evaluate the ‘penetrating power’ by enzymatic degradation of phospholipids with sPLA_2_s tested on densely packed monomolecular phospholipid film spread at the air–water interface. Crotoxin Subunit B, but not the complex, presented a high penetration capacity in monolayer films containing medium-chain negatively charged phospholipids [26]. It was proposed that the positive charge of Subunit B favors first recognition of the negative surface charge of procoagulant lipids, followed by a rapid penetration of the enzyme into the bilayer and resulting hydrolysis of procoagulant phospholipids. The same was not found for the CTX complex, proposing that the well-established role of Subunit A in preventing the CTX complex from unspecific binding to neuromuscular targets, and reduced Subunit B catalytic activity, increased the difficulty for the CTX complex in binding to a phospholipid–water interface [26,40,41]. 

Faure and colleagues [42] performed an interesting study, using in vitro enzymatic assays, surface plasma resonance, and molecular modeling in order to characterize the FXa-binding role of sPLA_2_ from the Viperidae family, including CTX and its subunits, and its effect on prothrombinase activity. As a first approach to screening for binding to FXa, the authors observed that the CTX complex is capable of binding to coagulation factor Xa. Notably, as previously reported, the toxin does not alter the activity of factor X alone [6], on which both reports align, indicating that binding occurs without compromising the factor’s functional integrity. Based on these findings, the authors proceeded to experiment using two distinct isoforms from subunit B (CBc and CBa_2_, which differ by 8 amino acids) and subunit A (isoform CA_2_). The CBc presented high binding affinity to FXa and a very high inhibitory effect on FXa-FVa-Ca^2+^ activity (prothrombinase complex without PL). Differently, CBa_2_ presented approximately 100x less efficiency on both binding and activity, whereas CA_2_ presented this neglected function. Interestingly, the carboximetilated form of CBc, without catalytic activity, showed no binding to FXa, suggesting that the proper conformation of the subunit is necessary for the interaction with the coagulation factor [42].

Using in silico predicted molecular models and docking, the authors of [42] observe that the molecular electrostatic potentials is positive in the front face of both isoforms CBc and CBa_2_, consisting of the anticoagulant region solvent-exposed parts of helix A, helix B, the Ca^2+^ loop, the helix C-β-wing loop, the front strand of the β-wing, and the C-terminal segment of the subunits. The regions of FXa at the interface with CBc and CBa_2_ include the N-terminal region of the EGF-like 2 domain in the light chain of FXa, as well as five regions in the heavy chain (consisting of the serine protease catalytic domain).

Apart from the prothrombin complex, Gimenez and colleagues [6] also demonstrated that CTX was capable of inhibiting extrinsic tenase complex activity. The toxin presented a high potency in inhibiting tissue factor (coagulation factor III) and factor VII complex’s capacity to activate coagulation factor X into FXa, higher when compared to the intrinsic tenase complex (FIX, FVIII, Ca^2+^ and PL) or prothrombinase complex (FXa, FVa, Ca^2+^ and PL). Another interesting finding demonstrated by the authors was that CTX was not capable of inhibiting isolated thrombin (activated factor II—FIIa) activity.

**Figure 3:**
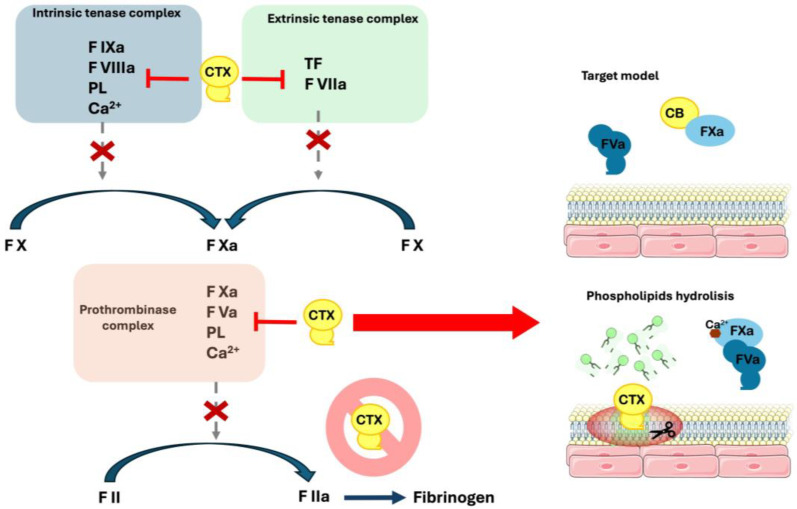
Anticoagulant mechanisms of Crotoxin. Schematic figure of Crotoxin’s capacity to interfere with the coagulation cascade. CTX complex is capable of inhibiting both the intrinsic (formed by the coagulation factors IXa—FIXa, VIIIa—FVIIIa, calcium—Ca^2+^ and phospholipid—PL) and extrinsic (coagulation factor VIIa -FVIIa- and tissue factor—TF) tenase complex, along with the prothrombinase complex (coagulation factors Xa—FXa, Va—FVa, calcium—Ca^2+^ and phospholipid—PL). On the prothrombinase complex, the toxin promotes inhibition by subunit B (CB) binding to FXa, and by PL hydrolysis. CTX complex is not capable of inducing coagulation factor II (FII—prothrombin) activation.

The above-mentioned overall results indicate that CTX is responsible for an anticoagulant activity mediated by PL disruption and coagulation factor binding mechanisms, and the toxin’s enzymatic activity is essential for this outcome. The catalytic active subunit B (CB) is responsible for the most potent anticoagulant behavior; however the whole complex sustains its capacity to inhibit prothrombinase, as well as intrinsic and extrinsic tenase complexes, demonstrating that subunit A (CA) modulates CB potency and might play an important role in limiting interaction with other biological targets (Figure 3).

### 3.3. Inhibition of Inflammation–Coagulation Crosstalk

Coagulation and inflammation are dynamically interconnected, forming a bidirectional feedback loop. The inflammatory response strongly promotes coagulation through endothelial cells and leukocyte activation and mediators’ release. These proinflammatory mediators are responsible for upregulating procoagulant factors, decreasing endogenous anticoagulant molecules, and reducing fibrinolysis. Together, these mechanisms create a prothrombotic state, linking immune defense with clot formation [43].

Crotoxin has been widely investigated concerning its immunomodulatory and anti-inflammatory effects. The toxin is responsible for modulating cellular events of both innate and humoral immunity, by producing endogenous lipid mediators with anti-inflammatory and immunosuppressive behavior, associated with pro-resolving mechanisms of immunological related disturbances [9]. Based on these properties, studies have been conducted in order to investigate the effect of CTX on modulating inflammation-induced coagulation.

Gimenez and colleagues [6] demonstrated that CTX was capable of reducing the production of proinflammatory cytokines IL-1β, IL-6, and TNF-α in lipopolysaccharide (LPS)-stimulated human mononuclear cells, as the toxin presented a more specific binding to monocytes. Consequently, a reduction in the expression of cell surface tissue factor (TF) was found. These same cells were submitted to a coagulation assay, incubating with platelet-poor plasma, and cells incubating with LPS only (higher surface TF expression) presented a procoagulant behavior, and when treated with CTX the procoagulant effect was impaired, considering the lesser expression of TF [6]. Tissue factor (coagulation factor III) is a transmembrane glycoprotein that forms the extrinsic tenase complex with FVIIa in order to trigger the coagulation cascade. Its expression is mediated by pathogenic agents, viruses, bacteria, fungi, and other agents such as snake venoms, mostly expressed by endothelial cells and monocytes [44,45].

In another approach involving coagulation–inflammation crosstalk, de Andrade and colleagues [8] have performed in vitro cell culture experiments evaluating the effect of CTX on LPS-stimulated endothelial cells’ expression of hemostatic-involved molecules. Cells pretreated with CTX prior to LPS stimulation were found to express less von Willebrand factor (vWF), responsible for mediating platelet adhesion to vascular injury sites, along with the stabilizing of circulating coagulation factor VIII, playing a critical role in hemostasis and vascular integrity [46]. Moreover, CTX was responsible for impairing the reduction production of Protein C from LPS-treated endothelial cells. Protein C is a vitamin K–dependent anticoagulant that inactivates factors Va and VIIIa, reducing clot formation [47]. Regarding the fibrinolysis pathway, the authors also assessed the expression of tissue plasminogen activator (t-PA), which converts plasminogen into plasmin to promote fibrin clot degradation, and plasminogen activator inhibitor-1 (PAI-1), which inhibits t-PA, thereby regulating fibrinolysis and maintaining hemostatic balance. The results demonstrated that LPS-stimulated cells showed an increase in PAI-1 and a decrease in t-PA activity, and CTX was capable of inhibiting these alterations.

**Figure 4:**
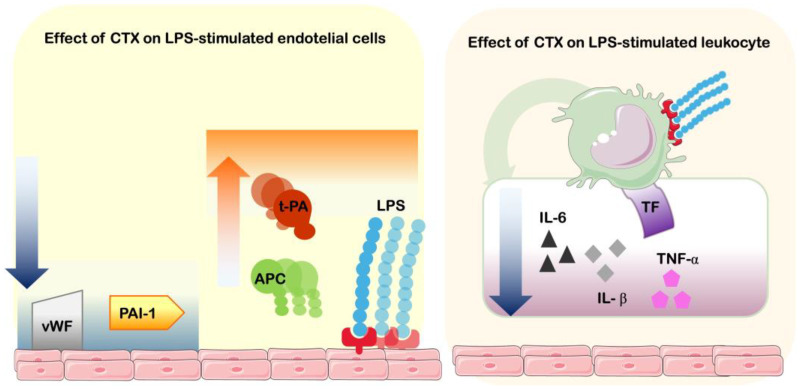
Effects of CTX on inflammation–coagulation crosstalk. Schematic figure of Crotoxin’s capacity to interfere with LPS-stimulation of endothelial cells and leukocytes. On endothelial cells, CTX induces the reduction of expression of von Willebrand factor (vWF) and plasminogen activator inhibitor-1 (PAI-1), and increases production of activated protein C (APC) and tissue plasminogen activator (t-PA). On blood mononuclear cells, CTX induces the reduction of cytokines interleukin-6 (IL-6), interleukin-1β (IL-1β) and tumor necrosis factor-α (TNF-α), as well as tissue factor (TF—coagulation factor III).

These results demonstrate that CTX is capable of reducing the hemostatic-related alterations induced by LPS, one of the most well-known agonists of inflammation, involving leukocytes and endothelial cells. The toxin was capable of impairing the dysregulation of procoagulant and anticoagulant factors, platelet aggregation mediators, and fibrinolytic pathways, in order to equilibrate the inflammatory-induced coagulation (Figure 4).

### 3.4. Modulation of Platelet Function

The investigation of CTX’s effects on platelets was first reported by Vargaftig and colleagues [48], and since then three other investigations have been performed [7,47,48]. The studies investigated both overlapping and distinct outcomes, employed diverse experimental protocols, and analyzed the effects of the CTX complex and its individual subunits on platelet aggregation.

Following the same pattern, three studies have investigated the functional aspects of the isolated subunits A and B. An agreement was found when authors demonstrated that both subunits (CA and CB), independently, demonstrated aggregation activity, using washed human platelets. In the case of CB, the authors described that the subunit was not able to induce aggregation, although it was found capable of inducing the production of arachidonic acid-derived thromboxanes A_2_ and B_2_ (TXA_2_ and TXB_2_); however, this was not sufficient for promoting platelet aggregation [46,47,48]. Regarding CA, the subunit not only present no platelet aggregation effect, but did not alter aggregation responses induced by arachidonic acid, platelet-activating factor (PAF), or thrombin in human platelet-rich plasma and washed platelets, nor stimulate TXB_2_ release or alter thrombin-induced TXB_2_ production [46,47,48].

**Figure 5:**
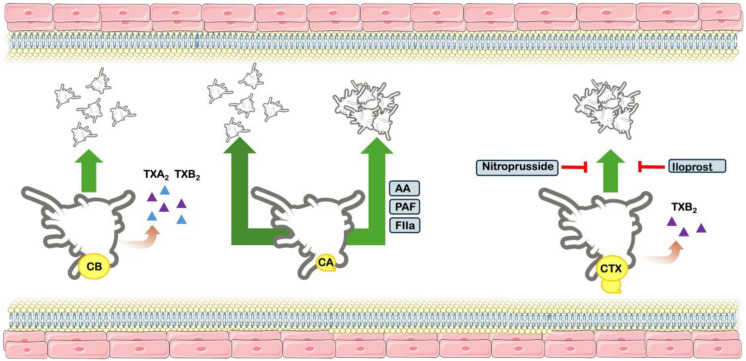
Effects on platelet function. Schematic figure of the effects of Crotoxin complex and subunits CB and CB on platelet function. CB does not induce platelet aggregation, although it is capable of producing both thromboxanes A_2_ and B_2_ (TXA_2_ and TXB_2_). CA does not induce platelet aggregation, and does not inhibit arachidonic acid (AA), platelet aggregation factor (PAF) and thrombin (FIIa). The CTX complex induces platelet aggregation, along with production of TXB_2_. The aggregation effect is inhibited by drugs Iloprost and nitroprusside.

However, Landucci and colleagues [7] observed that CTX complex is capable of inducing aggregation of washed human platelets. This effect was significantly inhibited by sodium nitroprusside and iloprost, known modulators of intracellular cGMP and cAMP pathways. Moreover, the authors observed that during platelet aggregation a substantial production of thromboxane B_2_ (TXB_2_) was found; however, pre-treatment with indomethacin, a cyclooxygenase inhibitor, drastically reduces TXB_2_ formation but not the platelet aggregation. Considering the results, authors have suggested that CTX-induced aggregation does not result from direct phospholipid hydrolysis, although arachidonic acid metabolites are produced, but rather from activation via specific intracellular signaling mechanisms [7] (Figure 5).

## 4. Envenoming Implications and Future Perspectives

CTX has been demonstrated as part of anticoagulant sPLA_2,_ along with other well-known toxins isolated from worldwide distributed snake venom, sharing similarities and differences. sPLA_2_ from *Vipera berus*, *Naja nigricollis*, *Vipera russelii*, and *Vipera ammodytes* are among the most frequently reported toxins involved in the modulation of hemostasis, although CTX remains the most extensively studied. Despite the limited number of comparative studies among these toxins, CTX continues to be the most widely investigated in terms of publication frequency and mechanistic insights (Table 1).

Although in vitro biochemical data provide extensive insights into the mechanisms underlying the anticoagulant activity of sPLA_2_, in vivo studies remain largely neglected. Most in vivo studies conducted with sPLA_2_, including CTX, have focused on other biological effects, such as a toxicological perspective (evaluation of neurotoxicity, myotoxicity and intravascular hemolysis) [54,55,56]. However, reports on the anticoagulant effect using animal models are limited to two sPLA_2_ isolated from *Daboia russelii* snake venom. Neupholipase, a neutral sPLA_2_, was responsible for prolonging tail bleeding and whole blood clotting time in mice injected with the toxin, without additional stimulus [57]. On the other hand, Daboxin P, a basic sPLA_2_, was capable of impairing vessel occlusion in a FeCl_3_-induced carotid artery thrombosis model [58].

Studies on the effects of anticoagulant sPLA_2_ using whole venom, instead of the isolated toxin, might not represent the best model of investigating its role in hemostasis, depending on the venom composition of snakes. In the cases of *Daboia russelii* and *Crotalus durissus* venoms, as examples, in vitro coagulation assays will present a procoagulant behavior, considering the presence of proteases in these venoms are capable of activating coagulation factors in order to trigger clotting [59,60]. Therefore, in these cases, the role of anticoagulant sPLA_2_ is suppressed. Moreover, the in vivo (animal models or clinical envenomation situations) contribution of both pro- and anticoagulant toxins in these venoms results in an increase in bleeding, since procoagulant toxins are responsible for consumption of coagulation factors. However, in the case of whole venoms in which anticoagulant activity of sPLA_2_ overlaps, such as in proper *Naja*, *Pseudoechis* and *Micrurus* species, in vitro plasma clotting assays demonstrate a prolonged clotting time [61,62,63]. In these cases, envenomation’s clinical aspects are followed by local and systemic coagulation disturbances, leading to bleeding events, which present a high similarity with procoagulant venoms that also contains anticoagulant sPLA_2_ [64]. These concepts reinforce the necessity for more in vivo studies with isolated anticoagulant sPLA_2_, as well as novel approaches using whole venoms in order to understand the role of anticoagulant sPLA_2_ on hemostasis, and its participation during envenomation.

Considering CTX’s consistent anticoagulant effects revised in the present review, novel in vivo prospects will not only improve data on the toxicological aspect of envenomation, but also open novel perspectives for possible drug-design with therapeutical applications on hemostatic disorders. This includes investigation of CTX on experimental models of coagulation disorders characterized by excessive clot formation, such as including deep vein thrombosis, pulmonary embolism, and atrial fibrillation-related thrombosis. Moreover, considering the toxin also presents an anti-inflammation behavior, inflammation-induced coagulation pathologies could be an interesting strategy. In sepsis, systemic inflammation induces disseminated intravascular coagulation (DIC) via mediator-driven endothelial activation, tissue factor expression, and platelet activation, leading to microvascular thrombosis and organ dysfunction [45]. CTX, presenting both anticoagulant and anti-inflammatory activities, could interrupt this pathological cycle: anti-inflammatory effects reduce cytokine release and endothelial injury, limiting coagulation initiation, while anticoagulant activity prevents clot propagation. By simultaneously targeting the inflammatory trigger and the coagulation cascade, such a dual-action therapy represents a promising novel strategy for managing sepsis-associated DIC.

Another interesting approach to anticoagulant sPLA_2_ studies consists of the use of enzymatic inhibitors. The literature has shown that available antivenom, along with new antibody formats and small molecule inhibitors, is capable of reducing anticoagulant activity of snake venoms, confirming its efficacy [65,66,67]. Among inhibitors, Varespladib, a small, synthetic molecule that broadly and potently inhibits phospholipases, has been assessed as a repositioning drug considering its previous development and tested for ulcerative colitis, rheumatoid arthritis, asthma, sepsis, and acute coronary syndrome, recently showing efficacy on sPLA_2_s [68]. Interestingly, Varespladib has been shown not only to inhibit *Crotalus durissus* lethality, but also the neuromuscular paralysis and myoticicity effects of the venom [69,70]. Regarding specifically CTX, previous reports have shown that Varesplabid was responsible for abrogating in vivo and in vitro myotoxic activity of CB [71], and neuromuscular blockade by CTX complex in phrenic nerve-diaphragm muscle preparations in mice [72]. The study of these inhibitors could be associated not only as another source of possible drug-design treatment for anticoagulant sPLA_2_ effects on snakebites, but also as an antidote strategy in future possible cases of a drug-design therapeutical approach for CTX, as an example.

## 5. Conclusions

CTXs’ role in hemostasis has been described in several scientific literature articles; however, proper attention has not been sufficiently paid. The present review performed a systematic description of all studies concerning toxin involvement in hemostatic events, and the mechanistic pathways involved. Like many other anticoagulant snake venom phospholipases A_2_, CTX is capable of directly inhibiting coagulation inhibition by interfering in different pathways of the coagulation cascade. The toxin hydrolyzes procoagulant phospholipids and inhibits coagulation factors, compromising tenase and prothrombinase complex activity and clot formation. Moreover, the toxin was capable of impairing inflammation-induced coagulation alterations, restoring/inhibiting the imbalance of endogenous factors.

Future perspectives should rely on improving data for a better understanding in vivo of the anticoagulant effects of CTX, avoiding toxic effects, and application for drug-design and as therapeutical applications for hemostatic disorders. Understanding its mechanisms may enable the development of innovative therapies for thrombosis, sepsis, and other hemostatic pathologies.

## Figures and Tables

**Table 1:** Anticoagulant mechanisms of isolated sPLA_2_ from snake venoms.

	*Crotalus durissus*			*Vipera berus*	*Naja nigricollis*	*Vipera russelii*
	CTX Complex	CA	CB	Vbb	CM-IV	RVAPLA2
Biochemical property	both	acid	basic	basic	basic	acid
Anticoagulant	moderate [6,26,38,39]	weak [26,38]	strong [26,38]	strong [26,49]	strong [26]	Strong [50]
Procoagulant phospholipid cleavage	Weak [6,26,40,41]	NA	strong [26]	strong [26,49]	weak [26]	strong [50,51]
Bind to FXa	Strong [42]	No binding [42]	strong [27,42]	weak [26]	strong [50]	strong [50]
FXa activity	invariant [6]	NA	NA	NA	NA	impair [50]
Prothrombinase complex activity	NA	weak [42]	strong [42]	weak impair [26]	strong [50,51]	NA
Intrinsic tenase complex activity	moderate [6]	NA	NA	NA	NA	NA
Extrinsic tenase complex activity	strong [6]	NA	NA	NA	strong [6]	NA
Factor IIa activity	invariant [6]	NA	NA	NA	invariant [52]	invariant [50]
Expression of cell-mediated hemostatic molecules	impair [6,8]	NA	NA	NA	NA	Pro-inflammatory [53]

NA = not assessed.

## Data Availability

No new data were created or analyzed in this study.

## Abbreviations

The following abbreviations are used in this manuscript:

CTX	Crotoxin
aSVPLA2	Anticoagulant phospholipases A_2_ from snake venoms
SVPLA_2_s	Snake venom phospholipases A_2_
PL	Phospholipids
FXa	Factor FXa
CA	Crotapotin
PT	Prothrombin time
aPTT	Activated partial thromboplastin time
CB	Catalytic active subunit B
TF	Cell surface tissue factor
vWF	von Willebrand factor
t-PA	Tissue plasminogen activator
TXB_2_	Thromboxane B_2_
